# Iron and the Breastfed Infant

**DOI:** 10.3390/antiox7040054

**Published:** 2018-04-06

**Authors:** James Friel, Wafaa Qasem, Chenxi Cai

**Affiliations:** 1 Department of Food and Human Nutritional Sciences, University of Manitoba, Winnipeg, MB R3T 2N2, Canada; caic3@myumanitoba.ca; 2 Food and Nutrition Administration, Ministry of Health, P.O. Box 4078, Safat 13041, Kuwait; qasemw@myumanitoba.ca

**Keywords:** iron, infant, mother’s milk

## Abstract

The first 6 months of life is a crucial time in meeting iron needs. The purpose of this review is to examine iron in mother’s milk and whether or not it meets the physiological needs of the growing infant. Key issues include iron content and iron transport from the mammary gland as well as when and what foods should be added to the solely breastfed infant. We examine these topics in light of new molecular biology findings in the mammary gland.

## 1. Introduction

Iron (Fe) is an essential nutrient that exists in low quantity in human milk. Whether or not iron in human milk will meet the developmental needs of the breastfed infant for the first 6 months of life is controversial [1,2]. Some countries recommend supplementation of iron as drops at 4 months, others do not [1,3,4]. This has bearing on the best time of introduction of solids as iron fortification of foods assists in meeting iron needs for the second half of infancy. Recent evidence has indicated that iron is not secreted into mother’s milk, and what iron that is present is present in low quantities. In terms of supplying solid foods that are rich in iron, there are new recommendations but no consensus. We review the evidence to support the early introduction of iron-enriched solid foods or iron drops by 4 months of age. We discuss these issues in light of recent molecular biological information derived from our studies of iron transporters in human mammary epithelial cells.

## 2. Iron 

### 2.1. Biological Functions of Fe

In the human body, iron is the most abundant trace element, and acts as a center for a broad spectrum of functions [4]. Its importance is derived from its redox activity, because iron exists mainly in ferrous (Fe^2+^) and ferric (Fe^3+^) oxidation states, which are interchangeable. The conversion of one state to the other forms part of the electron transport chain, essential in the generation of energy (ATP) during metabolism and in the reductions needed for synthesis [5]. There is little free iron in vivo. Iron is chelated to proteins or other molecules; this maintains solubility, limits participation in oxygen redox chemistry, and limits availability to microbes [6]. 

Proteins that bind iron act functionally as antioxidants [5,7]. An antioxidant is a substance that, when present at a low concentration compared with that of an oxidizable substrate, inhibits oxidation of the substrate, the process known as oxidative stress [5,7]. Iron is more known as an oxidant, a species that causes or promotes oxidation, whereas molecules that bind iron function as antioxidants. In contrast, vitamin C acts as a dietary iron reductant [5,7].

Hemoglobin (Hb) iron (60%), myoglobin iron (5%), both heme and non-heme enzymes (5%), and transferrin (less than 1%), are designated as functional iron. The remaining iron is found in the storage proteins ferritin (about 20%) and in hemosiderin (about 10%) [8]. The primary role of iron is for oxygen transport to assist in cell respiration. Myoglobin uses iron to store oxygen in muscle tissue. Enzyme iron functions in immune function and other metabolic actions [8]. An underappreciated role of iron is its importance in neurotransmitter function and myelination [9].

### 2.2. Fe Absorption and Transport

Fe uptake by enterocytes depends on the forms of the Fe. The sources of Fe include non-heme Fe, heme Fe, and ferritin. It has been proposed that heme Fe is transported by an intestinal heme transporter, heme carrier protein 1 (HCP1) [10], or another transporter [11]. This process has not yet been fully elucidated. Ferritin enters the enterocytes via an unknown mechanism, and it is likely then degraded in the lysosomes [12]. Non-heme ferric Fe must first be reduced to ferrous Fe by duodenal cytochrome b (DCYTB) [13] or other cell surface ferrireductases [14], and then be transported into the enterocytes by divalent metal transporter 1 (DMT1) [15]. Once it enters the cell, cellular Fe is stored as ferritin, used by the intracellular compartments, or exported into the blood for use by other tissues in the body [12]. Intracellular ferrous Fe is transferred across the basolateral membrane by ferroportin FPN [12], and is then oxidized to ferric Fe by hephaestin (HEPH), a membrane-anchored, multi-copper ferroxidase [16]. Then, ferric Fe binds to transferrin (TF) in the interstitial fluids and circulates throughout the body.

### 2.3. Fe Requirements of Infants 

At birth, full-term healthy infants have an iron content of about 75 mg/kg, with high blood volume, and Hb concentration in proportion to their body weight [17]. During the first few months of life, they experience a physiological decline in their blood volume and Hb concentration, and an active shift from fetal Hb to adult type Hb [18]. There are conflicting thoughts on the Fe requirement of infants. For newborn infants, most of the Fe in the body is found in Hb and some smaller stores. When a newborn is transferred from the uterine environment into an oxygen-rich atmosphere, the Hb level falls from 170 g/L to 120 g/L during the first 6 weeks of life [18]. For exclusively breastfed infants, the major source of Fe comes from body stores because the Fe content in human milk is extremely low [18,19]. Some researchers conclude that a normal healthy full-term infant has a sufficient amount of Fe until about 4 to 6 months of age [20]. Based on the average Fe concentration in human milk, the Institute of Medicine Dietary Reference Intakes (DRIs) for Fe for infants before the age of 6 months is 0.27 mg/d [21]. By contrast, the American Academy of Pediatrics recommends that exclusively breastfed, full-term infants receive 1 mg/kg per day of Fe supplement beginning at the age of 4 months [1]. 

### 2.4. Iron Content of Human Milk

As the current recommendations for infants are based on the iron content of human milk, we need to examine milk more closely. Human breast milk has very little iron (0.4 mg/L) [19]. Since breast milk is designed for humans by humans, it can be concluded that what is in breast milk is optimal for developing infants. While there is no controversy that iron in human milk is low, the current thought is that the small amount present must in some way be sufficient. Indeed, breast milk is thought to have “a special form” of iron often stated to be “highly bioavailable” [22,23]. To support this concept, Lonnerdal has shown the presence of a receptor in the infant gut specifically for lactoferrin-iron [24]. The concept that “breast milk is the perfect food” is challenged by the shortfall in several nutrients, including iron [25]. This is not a popular concept as a door might be opened for supplementation of any and all nutrients as is done for the premature infant [26]. Nonetheless, vitamin D is given to breastfed infants, although there is controversy around this as well [27]. 

The Fe content in human breast milk is considered low in comparison to maternal serum Fe. The Fe concentration in human colostrum is approximately 0.8 μg/mL; in mature breast milk, it is 0.2–0.4 μg/mL [28]. In contrast, the Fe concentrations in the milk of other species are much higher [28,29]. The Fe concentration in mature milk in rat is 5–10 μg/mL, which is about 25 times higher than the Fe concentration of human milk [29]. Although the Fe concentration is low in human milk, it is thought to be independent of the mother’s Fe status and it cannot be increased through maternal diet or Fe supplementation [30,31,32]. A recent study suggests the possibility that maternal iron status during pregnancy may affect the quantity of iron during lactation [33].

Human mammary epithelial cells do not secrete iron into breast milk. This surprising finding is based on the fact that the primary membrane iron exporter is not found in human lactating epithelial cells [34]. This accords with the prevailing view that body iron content is not controlled by excretion, but is controlled by absorption [35]. The iron content of human milk can be accounted in large amount by what is present in milk epithelial cells [19,36], which are the predominant cells in a healthy mother [37]. It may be that the infant gets all its needed iron from external sources as iron is ubiquitous, as it is the most common element on earth, and was readily available as contamination until recent times [38,39].

### 2.5. Supplementation with Iron Drops

The Fe status of exclusively breastfed infants and how it is maintained, remains a controversial topic in neonatal nutrition [40]. Some voices support Fe supplementation while others regard it as harmful due to potential Fe overload. Concerns have been expressed about Fe supplementation, as Fe is a potent pro-oxidant, and it cannot be actively excreted by humans [41]. Some studies have suggested that Fe supplementation may have adverse effects on iron-replete infants, including increased risk of infections and impaired growth [42]. Increased risk of severe infections seems to be restricted to malaria regions [43]. However, current evidence is not definitive, nor are the negative consequences of short-term impaired growth on long-term outcome. 

We have found that iron supplementation in early infancy improves cognitive development [44]. Healthy breastfed infants who received iron drops between 1 and 6 months of age had improvements in both psychomotor development skills and visual acuity, in comparison to control infants. From these findings, the American Academy of pediatrics recommended all exclusively breastfed infants should receive iron drops of 1 mg/kg/day at 4 months of age [22] to augment the low levels of iron in mother’s milk.

Results from a meta-analysis, which included four randomized control trials (RCT) involving 511 infants, supported early iron supplementation [45]. Current data suggests that though iron supplementation of healthy exclusively breastfed infants may improve their iron status and cognitive development, there may be a delay in their physical growth. There was no evidence to suggest iron supplementation could cause other adverse effects [45].

### 2.6. Iron Deficiency Anemia and Effects on Neurodevelopment

As iron supplements may be of benefit, maintaining an adequate level of Fe is critical to an infant’s physical and neurological development. Iron deficiency (ID) is commonly considered to develop in three stages: iron depletion, iron-deficient erythropoiesis, and iron deficiency anemia (IDA) [46]. 

Fe is critical for the rapid development of the central nervous system during infancy. Animal studies have shown that Fe is critical for many aspects of brain development, including myelination, monoamine neurotransmitter function, and neuronal and glial energy metabolism [41]. Some human studies have suggested that early ID is associated with later neurodevelopmental impairments. A longitudinal study in Costa Rica assessed changes in cognitive functioning after ID in infancy by evaluating four subsequent follow-ups at 5, 11–14, 15–18, and 19 years of age [47,48,49]. The results indicated that participants who had ID in infancy had lower cognitive scores over time compared to participants in the group with good Fe status. The results of these studies have increased concerns that early ID may irreversibly affect long-term neurodevelopment. A meta-analysis of 17 randomized clinical trials in children indicated that Fe supplements have positive cognitive effects in iron-deficient children [50]. Consequently, early prevention of ID seems to be extremely important. However, whether or not Fe has positive neurodevelopment effects on exclusively breastfed infants still needs to be clarified. 

### 2.7. Solids and Their Introduction

An important issue for the breastfed infant is the timing of the introduction of complementary feeding or solid foods. Once solids are started, the role of breast milk changes. Whereas breast milk was the only food the infant was receiving, the simplicity of the diet is now altered. Interactions between nutrients in different food items is now possible. When and what solids to introduce is controversial. Some [3] advocate delaying solids until 6 months of age, however, others suggest around 4 months is best [51,52]. In North America, the average age of introduction is around 4–5 months [53]. We think that there is some risk in being prescriptive about the timing of introduction of solids as some infants may or not need the energy and nutrients from solids at different times [52,54]. For health professionals to insist that 6 months is best for each and every infant is not biologically reasonable [55]. It is more reasonable for individual caregivers to base their decision of timing of introduction of solids on cues signifying physiological readiness provided by the infant [52]. It appears the main reason for recommending 6 months is an attempt to prolong the use of breastfeeding [4,54]. While a laudable goal, there are some infants who will need more nutrients than available from breast milk before 6 months of age [55].

### 2.8. Possible Adverse Effects of Fe in the Gastrointestinal Tract 

There is evidence that unabsorbed iron in the gastrointestinal tract will lead to the generation of reactive oxygen species [56,57]. This may be particularly true for infants consuming iron-fortified cereals of low bioavailability [58]. It is known that antioxidant foods, including fruit, may ameliorate the consequences of reactive oxygen species (ROS) generation [59]. We conducted a study in adults who received a large dose of iron concurrently with an antioxidant supplement [60]. Those subjects receiving the antioxidant supplement had significantly less ROS generation. Infants receiving iron-fortified cereals are consuming similar levels of iron that may subject them to inflammation [61]. 

### 2.9. Iron and Microbiota

The gastrointestinal tract is home to thousands of species of bacteria collectively known as the microbiome [62], which plays a role in metabolism. Dysbiosis of the infant microbiome may have unwanted consequences long-term [63]. The gut microbiome has been shown to be altered in animals receiving iron supplementation, however, little work has been done in infants [64,65,66]. We completed a study assessing the effect of meat and cereal based diets in infants on the infant gut microbiome [67]. Choice of first complimentary foods may influence gut inflammation and microbiota, potentially due to variations in iron absorption from different foods. Our findings supported the use of meats as a first food as suggested by Krebs group [66].

## 3. Complementary Feeding (CF)

There are few large studies examining complementary feeding of infants in developed countries. A large study in Canadian infants indicated that infants started solids as early as 4 months, little meat was consumed, and primary sources of iron were from iron-fortified infant cereals [53]. These findings do not indicate mother’s support of current recommendations to start solids at 6 months with meat as the first choice [3]. Mean iron intake from solids never met the 7- to 12-month recommendations for amounts of CF [53].

As there is little iron in mother’s milk [19], the low intake of iron from complementary foods becomes an issue. Iron in solids foods is found primarily in meat and fortified cereal, with very little found in fruits and vegetables [68]. Significant sources of iron, including meat, poultry, fish, and eggs, are not routinely consumed until 8–9 months of age [53]. In a large American study, infant cereal was the major source of most minerals for infants [69]. However, the form of iron used by manufacturers is of low bioavailability [70]. It is not surprising that 24% of Canadian infants have low iron stores [53]. Novel ways of meeting iron needs will have to be considered.

Further reviews on iron content of mother’s milk [18,19,23,28,29,30,32,33], iron metabolism [8,20,35,41], and timing of introduction of solid foods [52,54,55], address these issues as well.

## 4. Conclusions

(1) Is there enough iron in breast milk to meet infant needs for the first 6 months of life?
  Not enough to meet estimated needs.
(2) Will iron given as either drops or fortified foods before 6 months of age be harmful?
  Disturbed growth is not consistent, nor evidence of harm.
(3) When is the best time to introduce iron fortified solid foods?
  No earlier than 4 months, no later than 7 months, depending on the infant.
(4) What is the best solid food to give the infant first?
  Meat, no residual iron, to potentially induce ROS generation in gut.
(5) What is the best public health approach?
  Screen all infants at 4 months: otherwise, flag for any abnormality of infant or mother. There may be benefit in creating a composite system including ferritin, haemoglobin, mean corpuscular volume, dietary intake, and maternal risk factors. This would have to be examined carefully in a prospective cohort study.
